# Supplementary material for: Unique structural features in a deep-sea CYP51 relate to high pressure adaptation
Source: Res Sq. 2024 Dec 12:rs.3.rs-5589110. Preprint. [Version 1] doi: 10.21203/rs.3.rs-5589110/v1 (PMC11661291; doi:10.21203/rs.3.rs-5589110/v1)
Supplement: Supplement 1 [file NIHPPRS5589110V1-supplement-1.pdf]

Supplementary Files

This is a list of supplementary files associated with this preprint. Click to download.

- SupplementaryInformation.pdf
- 8sbifullvalidation.pdf
- 9batfullValidation.pdf
